# Data on the stated adoption decisions of Swiss farmers for variable rate nitrogen fertilization technologies

**DOI:** 10.1016/j.dib.2022.107979

**Published:** 2022-02-19

**Authors:** Karin Späti, Robert Huber, Ivana Logar, Robert Finger

**Affiliations:** a Agricultural Economics and Policy Group, Eidgenössiche Technische Hochschule Zürich, 8092 Zurich, Switzerland; b Department of Environmental Social Sciences, Eawag, Swiss Federal Institute of Aquatic Science and Technology, 8600 Dübendorf, Switzerland

**Keywords:** Precision agriculture, Variable rate technologies, Technology adoption, Choice modelling

## Abstract

We present data on the stated preference for the adoption of variable rate technologies from 418 crop farmers in Switzerland. The online survey was conducted online in spring 2021. It consisted of two parts: 1) a choice experiment and 2) questions about farmers' characteristics, expectations and beliefs, as well as their risk preferences. In the choice experiment, farmers were presented with eight consecutive choice tasks. Each task consisted of three alternatives, two hypothetical scenarios for variable rate technologies adoption and the status quo option. We used a split-sample approach and varied the additional profit margin gained through higher yields, label premiums or subsidies for one subsample (focussing on the willigness to accept) and additional cost (acquisition, maintenance and other costs) for the other subsample (focussing on the willigness to pay). Non-monetary attributes include 1) ownership of the technology; 2) potential to increase nitrogen use efficiency and thus reduce nitrogen losses to the environment; 3) uncertainties about the actual impact of the technology on yields and profits (reliability); 4) support in case of technical difficulties. We also collected data on farmers' experiences, attitudes and goals, as well as their risk preferences. Additionally, the survey data were matched with data from the cantonal farm census, which contains information on farm characteristics.

## Specifications Table

**Table:** 

Subject	Agricultural Economics
Specific subject area	Adoption of variable rate technologies in small-scaled farming systems
Type of data	CSV file
How the data were acquired	Online survey using Limesurvey combined with cantonal census data. Data cleaning with R
Data format	Raw Partly filtered (for reasons of confidentiality)
Description of data collection	The online questionnaire was distributed via Limesurvey to all crop farmers with more than 20% open cropland in the two cantons of Bern and Solothurn in Switzerland. A total of 418 farmers responded to the survey. Participation was incentivized. The data were anonymized.
Data source location	• Institution: ETH Zurich • City/Town/Region: Zurich • Country: Switzerland
Data accessibility	Repository name: ETH Zürich Research Collection Data identification number: 10.3929/ethz-b-000520042 Direct URL to data: https://www.research-collection.ethz.ch/handle/20.500.11850/520042

## Value of the Data

• The data can be used to elicit farmers' preferences for different characteristics of variable rate technologies. In addition, the dataset allows for a comparison of willingness to pay for and willingness to accept variable rate technologies. The data provides the basis for the analysis of behavioral factors that influence farmers’ adoption of variable rate technologies.
• The dataset will be important for researchers and policy makers who want to understand the factors driving farmers’ willingness to adopt variable rate technologies and assess policies aiming to support the uptake of such technologies.
• Data allows comparison of behavioral factors in the adoption decision of variable rate technologies as well as willingness to pay and willingness to accept across case studies and countries.
• Data can be used in in meta-analysis of farmers’ behavior and the assessment of theoretical models of farmers’ behavior.

## 1 Data Description

The dataset is stored in a CSV file (Survey_Data.csv) and contains information from an online survey, including a choice experiment, among Swiss farmers on their willingness to adopt technologies for site-specific nitrogen fertilization. The survey data contains information on factors influencing farmers' adoption decisions regarding the use of site-specific nitrogen fertilization technology, as well as information on farm and farmer's characteristics. The factors influencing farmers' adoption decisions were investigated through a discrete choice experiment. We used a split-sample approach to estimate the willingness to pay and willingness to accept for site-specific nitrogen fertilization. In addition, we elicited farmers’ risk preferences, environmental attitudes and technology affinity. This survey data is combined with census data on farm characteristics such as farm size. All personal information that may allow identifying individual farms (i.e., all qualitative data and personal comments) was removed from the dataset to protect confidentiality. Information on the internal validity of the data are provided in the supplementary material. The original questionnaire in German language (Survey_WTA_german.pdf, Survey_WTP_german.pdf), the English translation of the questionnaire (Survey_en.pdf), the dataset, and the codebook describing the variables (Description_Variables.pdf) are available on the ETH Zürich Research Collection. The survey was reviewed and approved by the ETH Ethics Committee (application no. EK 2021-N-14).

## 2 Experimental Design, Materials and Methods

**Fig. 1:**
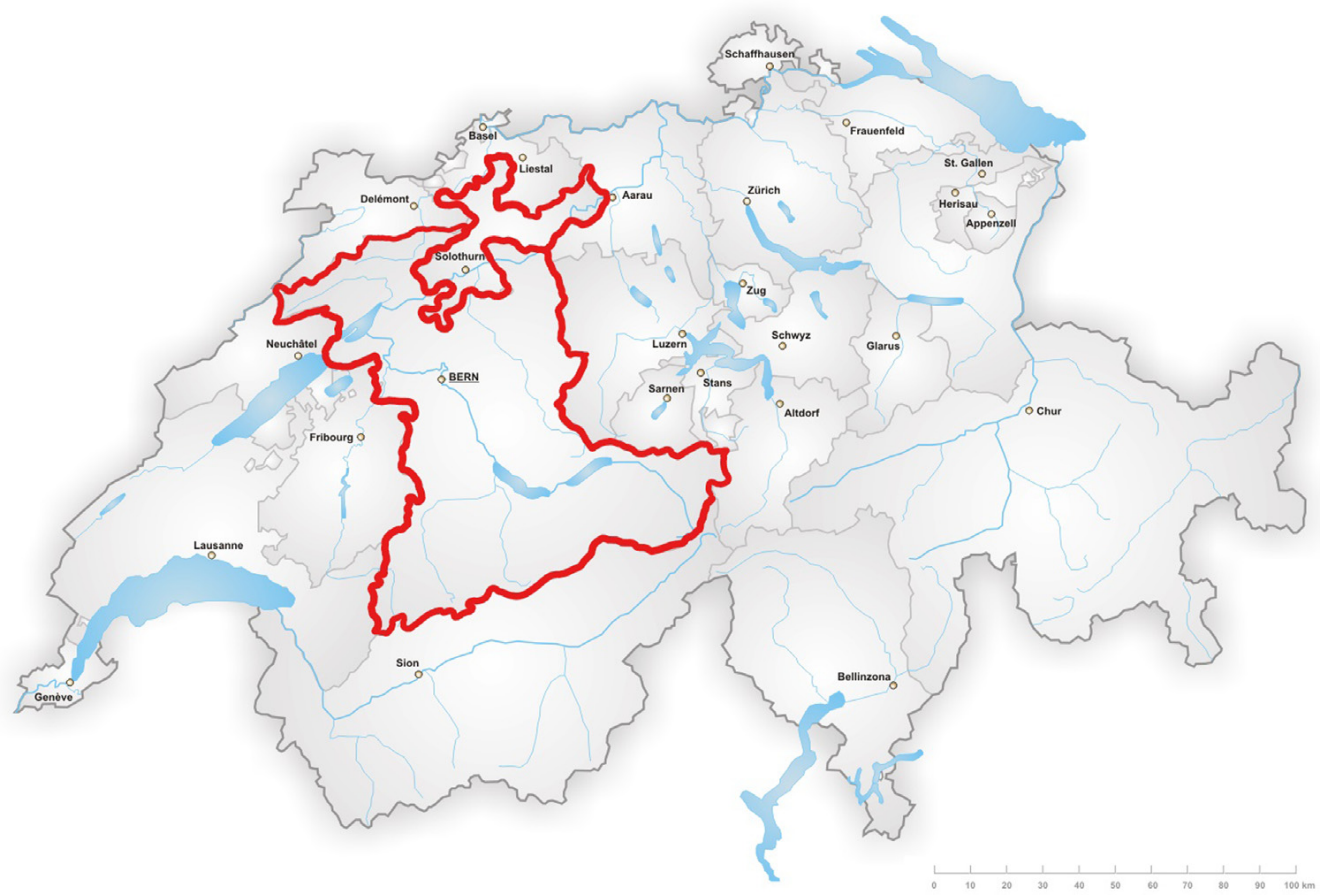
Map of Switzerland with the study area covering the cantons of Bern and Solothurn (red frame).

The survey was conducted using the online platform Limesurvey (www.limesurvey.org). The questionnaire was based on focus group discussions and pretested with students from an agricultural school. In March 2021, the online survey was sent by email to 4850 crop farmers in the Swiss cantons of Bern and Solothurn (Fig. 1).

We invited all farmers in these cantons with more than 20% open cropland to participate in the survey (selection based on census data). The invitation was sent by E-mail. The survey was online for two months. Farmers who did not complete the questionnaire received a reminder after three and six weeks. We received a total of 418 complete, valid responses, corresponding to a response rate of 8.74%. As an incentive to participate, farmers from the canton of Solothurn received a compensation of 30 CHF and farmers from the canton of Bern had the opportunity to win a voucher worth 100 CHF. In addition, interested farmers received a summary of the survey results. The survey contained 8 choice sets and 12 additional questions. The survey was divided into five sections and structured as follows:

1) Choice Experiment
2) Farmer's experiences, perceptions and preferences regarding technology and the environment
3) Risk attitudes
4) Social network
5) Characteristics of the farmer

### 2.1 Choice experiment

The first part of the survey consisted of a choice experiment. We used a split-sample approach and varied the monetary attribute, which was defined as the amount of additional profit margins gained through higher yields, label premiums or subsidies for one subsample (WTA) and as the additional cost (acquisition, maintenance and other costs) of the technology for the other subsample (WTP). The attributes for the choice experiment were identified based on a literature review [1], [2], [3]. This resulted in the following choice attributes: 1) Ownership of the technology, i.e., the farmer invests in the technology himself, along with other farmers, or uses the services through a contractor. 2) Potential to increase nitrogen use efficiency and thus reduce nitrogen loss to the environment [4], [5], [6], [7]. 3) Uncertainties about the actual impact of the technology on yields and usability also need to be considered, as this may influence farmers' decisions. These attributes were discussed in a focus group of five Swiss farmers. The focus group confirmed the relevance of the selected attributes, but also highlighted that the availability of technical support in the case of problems with the application can be an important factor in a farmer's adoption decisions. Therefore, we added another choice attribute in the experimental design to reflect the level of technical support, defined in terms of time needed to receive support Table 1. provides an overview of choice attributes and attribute levels used in the choice experiment.

**Table 1:** Choice attributes and attribute levels.

Attribute name	Description	Levels
Profit margin (WTA)/ Costs (WTP)	WTA: Additional profit margins resulting from the application of the technology. WTP: Additional costs due to the application of the technology.	100 CHF/ha and year 200 CHF/ha and year 300 CHF/ha and year 400 CHF/ha and year

Ownership	The farmer can either invest in the technology himself (self-investment), together with other farmers (joint investment) or purchase the service from a contractor	Self-investment Joint investment Contractor

Reduction of applied nitrogen	Reduction of applied nitrogen, without yield loss	No Reduction −20% −40%

Uncertainty	In how many out of five years does the technology bring a positive effect (+), no change (0) or a negative effect (−)	+++++ ++0+0 +++-+ 0+00+

Support	How long does it take for the farmer to receive support in the case of technical difficulties	No support Within 1h On the same day On the next day

**Fig. 2:**
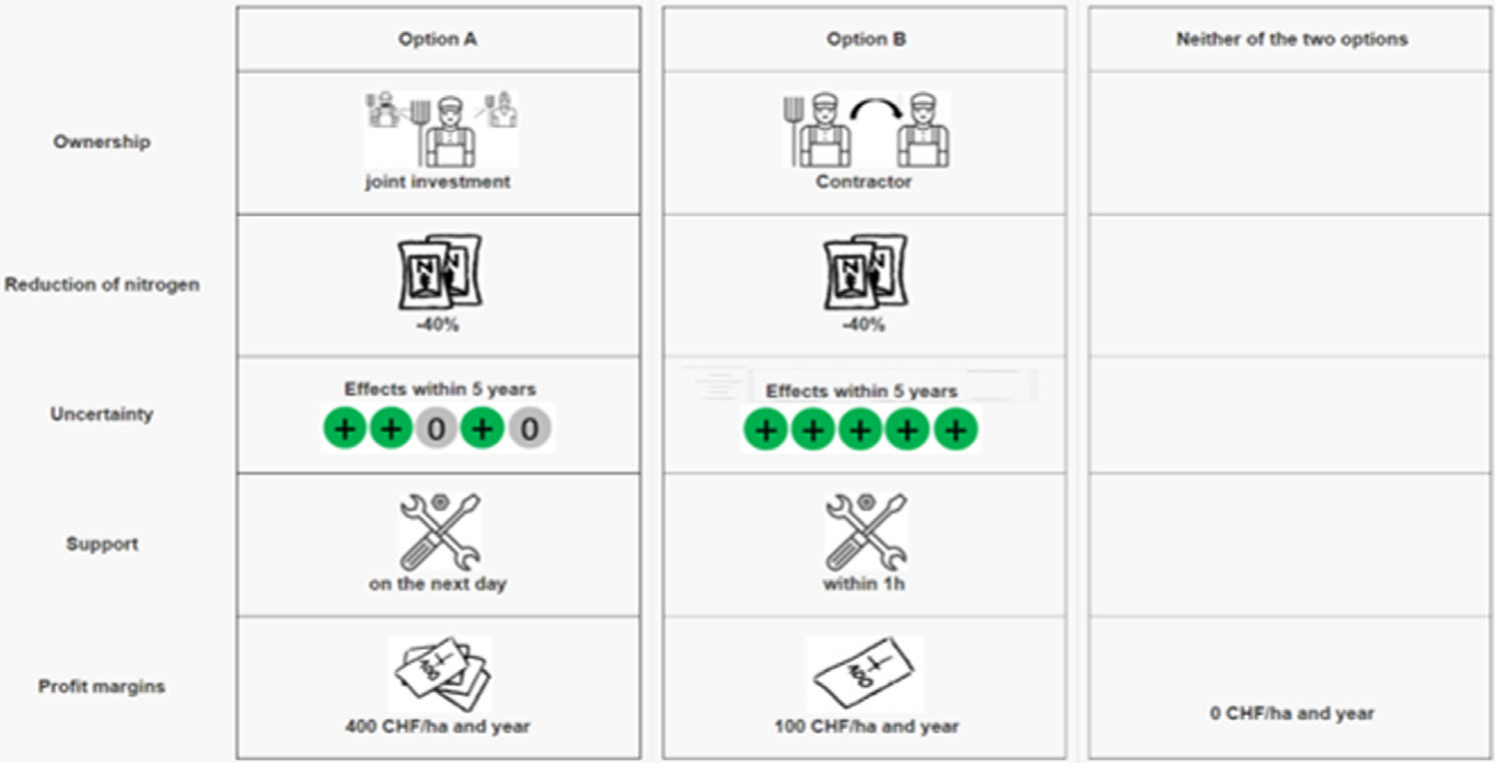
Example of a choice task.

The choice experiment design was generated in the Ngene software for each subsample. Priors from the survey pretest were used to generate the design for the final survey. The choice experiment design for each subsample consisted of eight choice tasks. In each choice task, the participants had to make a choice between three options. Two options represented different scenarios for the application of site-specific technologies. These options each differ with respect to five characteristics. The third option always represents the situation in which none of these technologies are applied (see Fig. 2 for an example). Each participant had to answer eight of these questions (CE1-8).

The questionnaire was pretested among 29 young farmers from an agricultural school. We revised the survey based on their feedback, which mainly suggested some linguistic adjustments to improve the comprehensibility of the choice experiment task.

### 2.2 Farmer's experiences, perceptions and attitudes

In the second part, the farmers were first asked whether they already have experience with site-specific fertilization (Q02) and if so, which technologies they already use on their farm. As site-specific technologies can be fairly complex, the provision of support could help to increase adoption. However, it is important to know from which source farmers prefer support. Therefore, we included a question about the preferred source of support in case of technical difficulties (Q03). High investment costs are expected to be a major obstacle to the adoption of variable rate technologies (VRT) [1]. Farmers who expect high costs for variable rate technologies adoption are probably less likely to adopt the technology. Therefore, we asked farmers about the expected costs of variable rate technologies adoption (Q04). To evaluate which measures could be used to promote the use of variable rate technologies, we also asked farmers how the use of the technology should increase the profit margins (Q05). In the second part, farmers were asked about their experience with variable rate technologies, preferred support for its application, level of investment costs, and preferred source of increase in contribution margins. In the next question, farmers had to indicate whether statements about VRT, its application, and the impact of the technology on them and their farm were true (Q06). The first two statements were about the impact of variable rate technologies on the environment and health, followed by statements about production goals and the innovativeness of farmers. Following the work of Kreft et al [8]. and Knapp et al [9], which was based on Abay et al [10] and Bandura [11], we included some questions on self-efficacy and locus of control. Finally, we asked the farmers whether it is important what others think of them and whether the decisions of other farmers have an effect on their decisions regarding the application of new technologies.

### 2.3 Risk attitudes

**Fig. 3:**
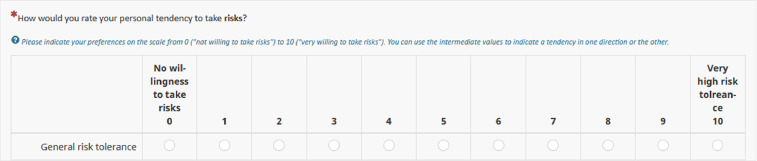
Example of risk assessment question.

To assess farmers' risk preferences, farmers were asked to rate their risk behavior on a Likert scale [12] (see Fig. 3 for an example).

First, they were asked about their risk preferences in general (Q07) and about their risk behavior in relation to the use of new technologies, agricultural production, and on-farm decisions (Q08). Next, they were asked how economically risky they consider it is to invest in new machines for site-specific nitrogen fertilization (Q09). Finally, farmers were asked to rate the importance of different aspects, such as changes in direct payment schemes, profitability, changes in product prices and contractors, in assessing the risk of investing in site-specific nitrogen application equipment (Q10).

### 2.4 Social network

According to Blasch et al [2], social networks can play a role in the use of precision technologies. Therefore, we asked farmers if they know any other farmers in their area, who already use site-specific nitrogen fertilization (Q11). If the question was answered yes, participants were asked to indicate the number of farmers who use variable rate technologies.

### 2.5 Characteristics of the farmer

**Fig. 4:**
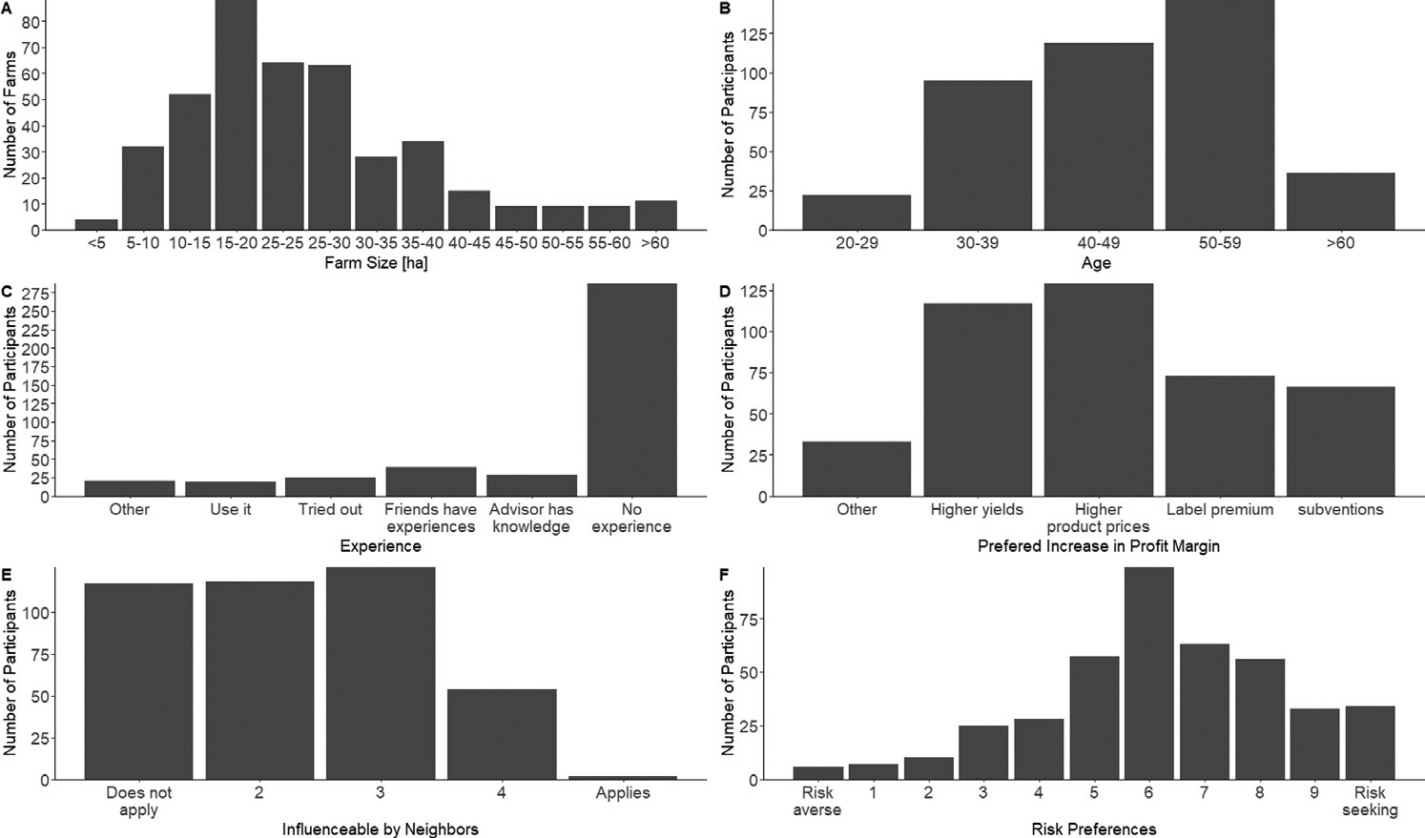
Exemplary summary graph for the distribution of participants into A) farm size groups and B) farmers’ age groups, C) experience with variable rate technologies, D) preferred form of increase in profit margins, E) whether farmers are influenced by their neighbors, and F) how farmers rate their personal risk propensities with respect to new technologies.

In the last part of the survey, participants were asked to indicate their year of birth (Q12) and the highest level of education they had achieved (Q13). The last question asked whether the succession of the farm had already been arranged (Q14) Fig. 4. gives an overview on the distribution of selected variables of the dataset.

## Ethics Statements

Participants had to give their consent for the data to be used anonymously for scientific purposes. The study was approved by the Ethics Commission of ETH Zurich (application no. EK 2021-N-14).

## CRediT Author Statement

**Karin Späti:** Conceptualization, Methodology, Formal analysis, Investigation, Data curation, Writing – original draft; **Robert Huber:** Conceptualization, Methodology, Writing - Review & Editing, Supervision; **Ivana Logar:** Conceptualization, Methodology, Writing – review & editing; **Robert Finger:** Conceptualization, Methodology, Writing – review & editing, Supervision.

## Declaration of Competing Interest

The authors declare that they have no known competing financial interests or personal relationships that could have appeared to influence the work reported in this paper.

## Data Availability

Data on the stated adoption decisions of Swiss farmers for variable rate nitrogen fertilization technologies (Original data) (ETH research collection).

## Appendix Supplementary materials

Image, application 1
