# Supplementary material for: Data on the stated adoption decisions of Swiss farmers for variable rate nitrogen fertilization technologies
Source: Data Brief. 2022 Feb 19;41:107979. doi: 10.1016/j.dib.2022.107979 (PMC8888958; doi:10.1016/j.dib.2022.107979)
Supplement: Supplementary file 1 [file mmc1.zip › Survey/Survey_en.pdf]

Declaration of consent

Your participation in the survey is voluntary. Your data and information will of course be treated as strictly confidential and will only be used anonymously for scientific purposes. Further information on the conditions of participation and data protection can be found here.
(This is a mandatory question)
□ I hereby confirm that my participation in the study is voluntary and that my data may be used.

I. Choice Experiment

CE1-8 Select one of the following options:

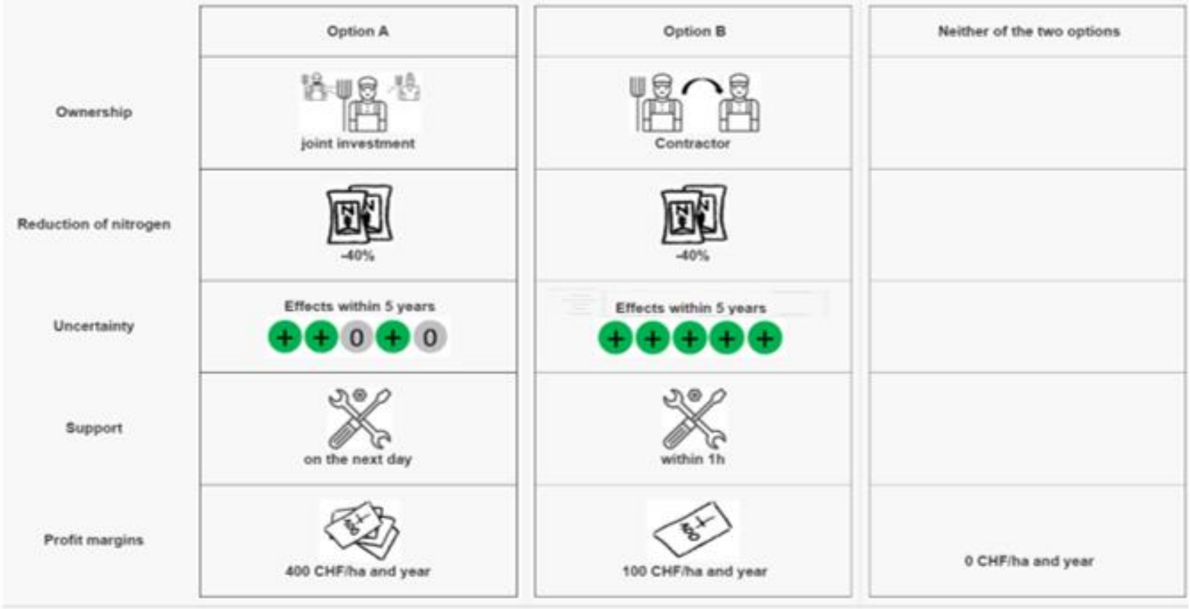

II. Perceptions and preferences

In the following, we would like to ask you some questions about your **personal perceptions and preferences** regarding **the application of site-specific nitrogen fertilisation**. It is about **your very personal perceptions and preferences** - so there are no right or wrong or better or worse answers. **All answers (as already mentioned above) are of course strictly anonymous and will only be evaluated in aggregated form.**

Q02 Do you already have experience with the application of site-specific nitrogen fertilisation? *
Please select one of the following answers:
- Yes, I already apply such technologies on my farm. If yes, which...
- Yes, I have already tried the application of such technologies on my farm.
- No, but I have neighbours/friends with experience.
- No, but my advisor knows.
- No, and I don't know anyone with experience.

Q03 When applying new technologies or technical problems, I prefer support and information from...
Please select the answers that apply:
- from cantonal advisory services
- other specialised agencies (e.g. Agroscope)

- from advisors of technology manufacturers
- from specialised media (newspapers/magazines/books/internet)
- from neighbouring farmers
- from farmer friends
- from the internet (e.g. blogs, online forums, etc.)
- Other:

Q04 What investment costs do you expect for the application of site-specific nitrogen fertilisation (per ha and year)?

	**0-50**	**51-100**	**101-150**	**151-200**	**201-250**	**251-300**	**301-350**	**351-400**	**401-450**	**451-500**
Investment cost in CHF per ha and year										

Q05 An increase in profit margins through the application of the technology should be based on...
Please select the answers that apply:
- Higher yields
- Higher production prices
- Higher product prices
- A label premium
- Subsidies
- Other:

Q06 The following statements apply to me and my farm:

	**Does not apply 1**	**2**	**3**	**4**	**Applies 5**
The application of site-specific nitrogen fertilisation will have positive effects on the environment.					
The application of site-specific nitrogen fertilisation will have positive effects on the health of farmers and consumers.					
I aim to use nitrogen as efficiently as possible.					
My nitrogen use depends on the price of wheat.					
My nitrogen use depends on nitrogen prices.					
Achieving high yields (dt/ha) is important to me.					
High biodiversity on my farmland is important to me.					
Achieving the highest possible income from production (incl. area contributions) is important to me.					
I am open to agricultural innovations and am often the first to implement them in the region.					

	Does not apply 1	2	3	4	Applies 5
When making important agricultural decisions, I often seek advice from my neighbours/colleagues.					
When I encounter difficulties in agricultural production, I can usually find a solution.					
Success in agricultural production depends mainly on the farmer's skills.					
I can solve most technical problems if I make an effort.					
Success in agricultural production can only be influenced to a small extent by farmers.					
It is important to reduce negative impacts of agricultural production on the environment.					
I like to learn about new technologies and their application.					
What my neighbours and friends think of me is important to me.					
Decisions made by neighbouring farmers influence my application decisions for technical innovations.					

III. Risk Attitudes

In the following, we would like to ask you some questions about your **risk attitudes**. It is about **your very personal perceptions and preferences** - so there are no right or wrong or better or worse answers. **All answers (as already mentioned above) are of course strictly anonymous and will only be evaluated in aggregated form.**

Q07 How would you rate your personal tendency to take risks?

	No willingness to take risks 0	1	2	3	4	5	6	7	8	9	Very high willingness to take risks 10
General risk tolerance											

Please indicate your preferences on the scale from 0 ("not willing to take risks") to 10 ("very willing to take risks"). You can use the intermediate values to indicate a tendency in one direction or the other.

Q08 How would you rate your personal tendency to take risks in the following areas?

	No willingness to take risks 0	1	2	3	4	5	6	7	8	9	Very high willingness to take risks 10
Application of new technologies											
Agricultural production											
Decisions on farm (general)											

Please indicate your preferences on the scale from 0 ("not willing to take risks") to 10 ("very willing to take risks"). You can use the intermediate values to indicate a tendency in one direction or the other.

Q09 How economically risky do you consider the following aspects to be with regard to investing in new machinery for site-specific nitrogen fertilisation?

	No risk 0	1	2	3	4	5	6	7	8	9	very high risk 10
Increased fluctuation of income											
Investment cannot be amortised											
Machines lose value quickly due to technical developments											

Q10 How strongly do the following statements contribute to your assessment of the risk of investing in machines for site-specific nitrogen application?

	Not important 1	2	3	4	Very important 5
I am afraid that direct payment programmes will soon change again.					
I am afraid that the technology will not lead to higher profit margins.					
I am afraid that the machine will not be used enough (acquisition not profitable).					
I am afraid that prices for arable crops will fall in the future.					

	Not important<br>1	2	3	4	Very important<br>5
Contractors will offer the service more cheaply and my investment will not be profitable.					

IV. Social network

Through the following questions, we aim to understand the role of social relations and networks in the application decision of new technologies, such as site-specific nitrogen fertilisation.

Q11 Are there farmers in your area who already use site-specific nitrogen fertilisation?
- Yes
- No
- Don't know

Q11a If yes, how many?

V. Personal characteristics of the farm manager and farm characteristics

In order to take into account differences between the farms in our results, we also would like to ask you in the following part of the survey about your personal characteristics - and characteristics of your farm. Your answers will be strictly anonymised and only evaluated in aggregated form.

Q12 What is the highest education level you have completed?
- Agricultural apprenticeship (EFZ)
- Agricultural master certificate
- Agricultural technician or agricultural commercial assistant (HF)
- University of Applied Sciences
- University or ETH
- Other qualifications

Q13 Has your farm succession already been arranged?
- Yes
- No
- Not yet relevant
