# Supplementary material for: Data on the stated adoption decisions of Swiss farmers for variable rate nitrogen fertilization technologies
Source: Data Brief. 2022 Feb 19;41:107979. doi: 10.1016/j.dib.2022.107979 (PMC8888958; doi:10.1016/j.dib.2022.107979)
Supplement: Supplementary file 1 [file mmc1.zip › Survey/Survey_WTA_german.pdf]

Umfrage zur teilflächenspezifischen Stickstoffdüngung

Liebe Landwirtinnen, liebe Landwirte,

in einem interdisziplinären Projekt der ETH Zürich (innofarm-projekt.org) untersuchen wir von der Gruppe für Agrarökonmie und -politik die Anwendung von Präzisionslandwirtschaftstechnologien in der Schweizer Landwirtschaft. Im Rahmen der folgenden Umfrage möchten wir untersuchen, welche Faktoren die Anwendungsentscheidungen von solchen Technologien beinflussen. Die Umfrage erfolgt in Zusammenarbeit mit:

SOBV Solothurner Bauernverband KANTON solothurn Kanton Bern Canton de Berne

Die Verbesserung der Effizienz der Stickstoffnutzung ist entscheidend für die Bewältigung aktueller Herausforderungen in der Landwirtschaft. Durch die zielgerichtete (Menge und Ort) Ausbringung von Stickstoff können Präzisionslandwirtschaftstechnologien dazu beitragen Stickstoff effizienter zu nutzen und Verluste zu verringern. Im Folgenden nennen wir diese Technologien "teilflächenspezifische Stickstoffdüngung". Zur Gewinnung von Informationen zur teilflächenspezifischen Stickstoffbedarfsermittlung und Düngerausbringnung stehen dabei verschiedene Methoden (z.B. basierend auf Satellitenaufnahmen, Sensoren an Traktoren, oder Kameras an Drohnen) zur Verfügung, die sich unter anderem in ihren Kosten unterscheiden. Obwohl diese Präzisionslandwirtchaftstechnologien bereits seit einigen Jahren verfügbar sind, ist die Anwendungsrate in der Schweiz eher gering. Im Rahmen dieser Umfrage möchten wir herausfinden, welche Faktoren die Anwendungsentscheidung beeinflussen.

Die Umfrage ist gegliedert in fünf Abschnitte: 1) einem Choice Experiment, sowie Fragen zu 2) Wahrnehmungen und Präferenzen in Bezug auf die Anwendungen von neuen Technologien, 3) Risikoeinstellungen, 4) Ihrem sozialen Netzwerk und 5) Ihrer Person und Ihrem Betrieb. Die Befragung dauert 20-30 Minuten.

Wir bedanken uns ganz herzlich für Ihre Teilnahme!

Bei Fragen oder Unklarheiten wenden Sie sich bitte an:

Karin Späti

Gruppe für Agrarökonomie und -politik, ETH Zürich

kspaeti@ethz.ch

In dieser Umfrage sind 34 Fragen enthalten.

Einverständniserklärung

Ihre Teilnahme an der Umfrage ist freiwillig. Ihre Daten und Angaben werden selbstverständlich streng vertraulich behandelt und ausschliesslich anonymisiert zu wissenschaftlichen Zwecken verwendet. Weitere Informationen zu Teilnahmebedingnung und Datenschutz erhalten Sie hier.

*

Bitte wählen Sie nur eine der folgenden Antworten aus:

◯ Hiermit bestätige ich, dass meine Teilnahme an der Studie freiwillig ist und meine Daten verwendet werden dürfen.

Beschreibung der Umfrage

Im ersten Teil der Befragung geht es darum, dass Sie unterschiedliche Varianten einer teilflächenspezifischen Stickstoffdüngung* gegeneinander abwägen. Sie müssen jeweils eine Wahl zwischen drei Varianten treffen. Zwei Varianten repräsentieren verschiedene Szenarien zur Anwendung teilflächenspezifischer Technologien. Diese Varianten unterscheiden sich jeweils in Bezug auf fünf Eigenschaften, welche in der folgenden Tabelle ausführlich beschrieben sind. Die dritte Variante repräsentiert die Situation, in welcher keine dieser Technologien angewendet wird.

Vergleichen Sie die drei Varianten und überlegen Sie sich, welche Kombination dieser Eigenschaften für Sie persönlich eher in Frage käme. Wenn Sie eine Variante bevorzugen, dann markieren Sie diese, indem Sie das Kästchen unter der entsprechenden Variante anklicken. Falls für Sie und Ihren Betrieb keine der beiden Alternativen sinnvoll erscheint, klicken Sie auf das Kästchen «keine der beiden Varianten». Damit wir Informationen zu den unterschiedlichen Eigenschaften sammeln können, müssen Sie sich 8-mal entscheiden. Wählen Sie jeweils ihre bevorzugte Variante aus und klicken Sie auf weiter. Eine ausführlichere Anleitung dazu finden Sie in diesem Video.

Auf der folgenden Seite finden Sie eine detaillierte Beschreibung der Eigenschaften. Bitte lesen Sie diese aufmerksam durch. In jeder der Varianten werden diese unterschiedlich zusammengesetzt sein.

*zielgerichtete Ausbringnung von Stickstoff, basierend auf Informationen, welche über Satellitenbilder, Drohnenaufnahmen, Stickstoffsensoren an Landmaschinen etc. gewonnen werden.

Beschreibung der Technologieeigenschaften

Eigentümerstruktur

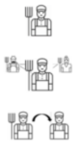

Es gibt verschiedene Möglichkeiten, wie Sie die Technologie anschaffen können. Sie könnten eine **Eigeninvestition** bevorzugen. Dabei würden Sie die Technologie selbst kaufen und besitzen. Die zweite Möglichkeit wäre eine gemeinsame **Investition mit anderen Betrieben** zusammen. Die Technologie müsste dann auch gemeinsam genutzt werden. Eine dritte Möglichkeit wäre der Bezug der Leistung von einem **Lohnunternehmer**, dabei erfolgt keine eigene Investition in die Technologie. Die Kosten würden dann nur für die Dienstleistung anfallen.

Stickstoffreduktion

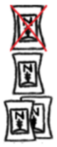

Die teilflächenspezifische Stickstoffdüngung verspricht einen effizienteren Stickstoffeinsatz. Ein Zielertrag kann/soll dabei mit einem reduzierten Sticksstoffeinsatz realisiert werden. Überlegen Sie sich, wie wichtig das Ausmass der möglichen Reduktion der ausgebrachten Stickstoffmengen ist, damit Sie die Technologie einsetzen würden.

Unsicherheit

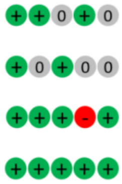

Es kann sein, dass die Technologie aufgrund von Wetterbedingnungen oder Messfehlern nicht in jedem Jahr den erwarteten Nutzen erbringt. Es gibt daher eine gewisse Unsicherheit, ob der gewünschte Nutzen in Form von Mehrerlösen oder tieferen Kosten in jedem Jahr erreicht wird oder nicht. Dieses Element wird folgendermassen dargestellt: Sie sehen jeweils eine kleine Tabelle, welche sich über fünf Jahre erstreckt. Für jedes Jahr ist eingezeichnet, ob die Technologie zum erwarteten Erlös führt (+) oder ob für dieses Jahr keine zusätzlichen Erlöse erzielt werden (0) oder ob sogar ein Verlust entsteht (-). Überlegen Sie sich jeweils, ob Sie bereite wären, diese Unsicherheit in der entsprechenden Variante mitzutragen.

Unterstützung

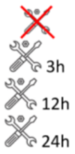

Technologie funktioniert nicht immer. Dieses Element beschreibt, wie lange es dauert, bis Sie bei technischen Schwierigkeiten oder anderen Problemen bei der Anwendung der Technologie Unterstützung erhalten. Woher diese Unterstützung kommt ist für die Wahl der Variante in unserem Fragebogen nicht relevant. Überlegen Sie sich nur, ob Sie bereite wären, die Technologie einzusetzen auch wenn es zu Verzögerungen in der Anwendung kommen kann, weil die Maschinen kurzfristig nicht einsetzbar sind.

Höhere Deckungsbeiträge

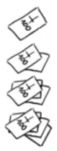

Durch die Anwendung der teilflächenspezifischen Stickstoffdüngnung können höhere Deckungsbeiträge erzielt werden, beispielsweise über höhere Ernteerträgen oder Labelprämien aber auch zusätzlichen Direktzahlungen. Die jeweils angegeben Beträge gelten unabhängig davon, ob Sie die Technologie allein oder gemeinsam mit Anderen nutzen/besitzen. **Überlegen Sie sich jeweils, wie stark sich die Deckungsbeiträge erhöhen müssten, damit Sie die Technologie einsetzen würden.**

I. Choice Experiment

Wählen Sie eine der folgenden Optionen: *

Bitte wählen Sie die zutreffende Antwort für jeden Punkt aus:

	Variante A	Variante B	Keine der beiden Varianten
Eigentümerstruktur	gemeinsame Investition	Lohnunternehmer	
Stickstoffeinsparung	-40%	-40%	
Unsicherheit	Auswirkungen während 5 Jahren<br>+ + 0 + 0	Auswirkungen während 5 Jahren<br>+ + + + +	
Unterstützung	am nächsten Tag	innerhalb 1 Stunde	
Erhöhung Deckungsbeitrag	400 CHF/ha	100 CHF/ha und Jahr	0 CHF/ha und Jahr
	○	○	○

Eigentümerstruktur	Stickstoffreduktion	Unsicherheit	Unterstützung	Erhöhung Deckungsbeitrag
Eigeninvestition	Keine Reduktion	+ + 0 + 0	Keine Unterstützung	100 CHF/ha und Jahr
Lohnunternehmer	-20%	+ 0 + 0 0	innerhalb 1 Stunde	200 CHF/ha und Jahr
Gemeinsame Investition	-40%	+ + + - +	am selben Tag	300 CHF/ha und Jahr
		+ + + + +	am nächsten Tag	400 CHF/ha und Jahr
Der Landwirt kann entweder selbst in die Technologie investieren (Eigeninvestition), zusammen mit anderen Landwirten (gemeinsames Investition) oder die Leistung von einem Lohnunternehmer beziehen.	Reduktion der ausgebrachten Stickstoffmenge, ohne Ertragsverlust.	In wie vielen aus fünf Jahren bringt die Technologie einen Nutzen (+), keine Veränderung(0) oder einen Verlust (-)	Wie lange dauert es bis der Landwirt bei technischen Schwierigkeiten Unterstützung erhält.	Durch die Anwendung der Technologie entstehende höhere Deckungsbeiträge.

Wählen Sie eine der folgenden Optionen: *

Bitte wählen Sie die zutreffende Antwort für jeden Punkt aus:

	Variante A	Variante B	Keine der beiden Varianten
Eigentümerstruktur	Eigenivestition	gemeinsame Investition	
Stickstoffeinsparung	-20%	Keine Reduktion	
Unsicherheit	Auswirkungen während 5 Jahren<br>+ + 0 + 0	Auswirkungen während 5 Jahren<br>+ + + + +	
Unterstützung	keine Unterstützung	Keine Unterstützung	
Erhöhung Deckungsbeitrag	100 CHF/ha	400 CHF/ha und Jahr	0 CHF/ha und Jahr
	○	○	○

Eigentümerstruktur	Stickstoffreduktion	Unsicherheit	Unterstützung	Erhöhung Deckungsbeitrag
Eigeninvestition	Keine Reduktion	+ + 0 + 0	Keine Unterstützung	100 CHF/ha und Jahr
Lohnunternehmer	-20%	+ 0 + 0 0	innerhalb 1 Stunde	200 CHF/ha und Jahr
Gemeinsame Investition	-40%	+ + + - +	am selben Tag	300 CHF/ha und Jahr
		+ + + + +	am nächsten Tag	400 CHF/ha und Jahr
Der Landwirt kann entweder selbst in die Technologie investieren (Eigeninvestition), zusammen mit anderen Landwirten (gemeinsames Investition) oder die Leistung von einem Lohnunternehmer beziehen.	Reduktion der ausgebrachten Stickstoffmenge, ohne Ertragsverlust.	In wie vielen aus fünf Jahren bringt die Technologie einen Nutzen (+), keine Veränderung(0) oder einen Verlust (-)	Wie lange dauert es bis der Landwirt bei technischen Schwierigkeiten Unterstützung erhält.	Durch die Anwendung der Technologie entstehende höhere Deckungsbeiträge.

Wählen Sie eine der folgenden Optionen: *

Bitte wählen Sie die zutreffende Antwort für jeden Punkt aus:

	Variante A	Variante B	Keine der beiden Varianten
Eigentümerstruktur	Lohnunternehmer	gemeinsame Investition	
Stickstoffeinsparung	-40%	-20%	
Unsicherheit	Auswirkungen während 5 Jahren<br>+ + + - +	Auswirkungen während 5 Jahren<br>+ + + - +	
Unterstützung	keine Unterstützung	am selben Tag	
Erhöhung Deckungsbeitrag	200 CHF/ha	100 CHF/ha und Jahr	0 CHF/ha und Jahr
	○	○	○

Eigentümerstruktur	Stickstoffreduktion	Unsicherheit	Unterstützung	Erhöhung Deckungsbeitrag
Eigeninvestition	Keine Reduktion	+ + 0 + 0	Keine Unterstützung	100 CHF/ha und Jahr
Lohnunternehmer	-20%	+ 0 + 0 0	innerhalb 1 Stunde	200 CHF/ha und Jahr
Gemeinsame Investition	-40%	+ + + - +	am selben Tag	300 CHF/ha und Jahr
		+ + + + +	am nächsten Tag	400 CHF/ha und Jahr
Der Landwirt kann entweder selbst in die Technologie investieren (Eigeninvestition), zusammen mit anderen Landwirten (gemeinsames Investition) oder die Leistung von einem Lohnunternehmer beziehen.	Reduktion der ausgebrachten Stickstoffmenge, ohne Ertragsverlust.	In wie vielen aus fünf Jahren bringt die Technologie einen Nutzen (+), keine Veränderung(0) oder einen Verlust (-)	Wie lange dauert es bis der Landwirt bei technischen Schwierigkeiten Unterstützung erhält.	Durch die Anwendung der Technologie entstehende höhere Deckungsbeiträge.

Wählen Sie eine der folgenden Optionen: *

Bitte wählen Sie die zutreffende Antwort für jeden Punkt aus:

	Variante A	Variante B	Keine der beiden Varianten
Eigentümerstruktur	gemeinsame Investition	Lohnunternehmer	
Stickstoffeinsparung	-40%	-40%	
Unsicherheit	Auswirkungen während 5 Jahren<br>+ 0 + 0 0	Auswirkungen während 5 Jahren<br>+ + 0 + 0	
Unterstützung	innerhalb einer Stunde	am selben Tag	
Erhöhung Deckungsbeitrag	200 CHF/ha	300 CHF/ha und Jahr	0 CHF/ha und Jahr
	○	○	○

Eigentümerstruktur	Stickstoffreduktion	Unsicherheit	Unterstützung	Erhöhung Deckungsbeitrag
Eigeninvestition	Keine Reduktion	+ + 0 + 0	Keine Unterstützung	100 CHF/ha und Jahr
Lohnunternehmer	-20%	+ 0 + 0 0	innerhalb 1 Stunde	200 CHF/ha und Jahr
Gemeinsame Investition	-40%	+ + + - +	am selben Tag	300 CHF/ha und Jahr
		+ + + + +	am nächsten Tag	400 CHF/ha und Jahr
Der Landwirt kann entweder selbst in die Technologie investieren (Eigeninvestition), zusammen mit anderen Landwirten (gemeinsames Investition) oder die Leistung von einem Lohnunternehmer beziehen.	Reduktion der ausgebrachten Stickstoffmenge, ohne Ertragsverlust.	In wie vielen aus fünf Jahren bringt die Technologie einen Nutzen (+), keine Veränderung(0) oder einen Verlust (-)	Wie lange dauert es bis der Landwirt bei technischen Schwierigkeiten Unterstützung erhält.	Durch die Anwendung der Technologie entstehende höhere Deckungsbeiträge.

Wählen Sie eine der folgenden Optionen: *

Bitte wählen Sie die zutreffende Antwort für jeden Punkt aus:

	Variante A	Variante B	Keine der beiden Varianten
Eigentümerstruktur	Eigeninvestition	Lohnunternehmer	
Stickstoffeinsparung	-20%	Keine Reduktion	
Unsicherheit	Auswirkungen während 5 Jahren<br>+ + + + +	Auswirkungen während 5 Jahren<br>+ + 0 + 0	
Unterstützung	am selben Tag	innerhalb 1 Stunde	
Erhöhung Deckungsbeitrag	300 CHF/ha	300 CHF/ha und Jahr	0 CHF/ha und Jahr
	○	○	○

Eigentümerstruktur	Stickstoffreduktion	Unsicherheit	Unterstützung	Erhöhung Deckungsbeitrag
Eigeninvestition	Keine Reduktion	+ + 0 + 0	Keine Unterstützung	100 CHF/ha und Jahr
Lohnunternehmer	-20%	+ 0 + 0 0	innerhalb 1 Stunde	200 CHF/ha und Jahr
Gemeinsame Investition	-40%	+ + + - +	am selben Tag	300 CHF/ha und Jahr
		+ + + + +	am nächsten Tag	400 CHF/ha und Jahr
Der Landwirt kann entweder selbst in die Technologie investieren (Eigeninvestition), zusammen mit anderen Landwirten (gemeinsames Investition) oder die Leistung von einem Lohnunternehmer beziehen.	Reduktion der ausgebrachten Stickstoffmenge, ohne Ertragsverlust.	In wie vielen aus fünf Jahren bringt die Technologie einen Nutzen (+), keine Veränderung(0) oder einen Verlust (-)	Wie lange dauert es bis der Landwirt bei technischen Schwierigkeiten Unterstützung erhält.	Durch die Anwendung der Technologie entstehende höhere Deckungsbeiträge.

Wählen Sie eine der folgenden Optionen: *

Bitte wählen Sie die zutreffende Antwort für jeden Punkt aus:

	Variante A	Variante B	Keine der beiden Varianten
Eigentümerstruktur	Eigeninvestition	Eigeninvestition	
Stickstoffeinsparung	Keine Einsparung	-20%	
Unsicherheit	Auswirkungen während 5 Jahren<br>+ 0 + 0 0	Auswirkungen während 5 Jahren<br>+ + + - +	
Unterstützung	am selben Tag	am nächsten Tag	
Erhöhung Deckungsbeitrag	300 CHF/ha	400 CHF/ha und Jahr	0 CHF/ha und Jahr
	○	○	○

Eigentümerstruktur	Stickstoffreduktion	Unsicherheit	Unterstützung	Erhöhung Deckungsbeitrag
Eigeninvestition	Keine Reduktion	+ + 0 + 0	Keine Unterstützung	100 CHF/ha und Jahr
Lohnunternehmer	-20%	+ 0 + 0 0	innerhalb 1 Stunde	200 CHF/ha und Jahr
Gemeinsame Investition	-40%	+ + + - +	am selben Tag	300 CHF/ha und Jahr
		+ + + + +	am nächsten Tag	400 CHF/ha und Jahr
Der Landwirt kann entweder selbst in die Technologie investieren (Eigeninvestition), zusammen mit anderen Landwirten (gemeinsames Investition) oder die Leistung von einem Lohnunternehmer beziehen.	Reduktion der ausgebrachten Stickstoffmenge, ohne Ertragsverlust.	In wie vielen aus fünf Jahren bringt die Technologie einen Nutzen (+), keine Veränderung(0) oder einen Verlust (-)	Wie lange dauert es bis der Landwirt bei technischen Schwierigkeiten Unterstützung erhält.	Durch die Anwendung der Technologie entstehende höhere Deckungsbeiträge.

Wählen Sie eine der folgenden Optionen: *

Bitte wählen Sie die zutreffende Antwort für jeden Punkt aus:

	Variante A	Variante B	Keine der beiden Varianten
Eigentümerstruktur	Lohnunternehmer	Lohnunternehmer	
Stickstoffeinsparung	Keine Einsparung	-20%	
Unsicherheit	Auswirkungen während 5 Jahren<br>+ + + + +	Auswirkungen während 5 Jahren<br>+ 0 + 0 0	
Unterstützung	am nächsten Tag	Keine Unterstützung	
Erhöhung Deckungsbeitrag	100 CHF/ha	200 CHF/ha und Jahr	0 CHF/ha und Jahr
	○	○	○

Eigentümerstruktur	Stickstoffreduktion	Unsicherheit	Unterstützung	Erhöhung Deckungsbeitrag
Eigeninvestition	Keine Reduktion	+ + 0 + 0	Keine Unterstützung	100 CHF/ha und Jahr
Lohnunternehmer	-20%	+ 0 + 0 0	innerhalb 1 Stunde	200 CHF/ha und Jahr
Gemeinsame Investition	-40%	+ + + - +	am selben Tag	300 CHF/ha und Jahr
		+ + + + +	am nächsten Tag	400 CHF/ha und Jahr
Der Landwirt kann entweder selbst in die Technologie investieren (Eigeninvestition), zusammen mit anderen Landwirten (gemeinsames Investition) oder die Leistung von einem Lohnunternehmer beziehen.	Reduktion der ausgebrachten Stickstoffmenge, ohne Ertragsverlust.	In wie vielen aus fünf Jahren bringt die Technologie einen Nutzen (+), keine Veränderung(0) oder einen Verlust (-)	Wie lange dauert es bis der Landwirt bei technischen Schwierigkeiten Unterstützung erhält.	Durch die Anwendung der Technologie entstehende höhere Deckungsbeiträge.

Wählen Sie eine der folgenden Optionen: *

Bitte wählen Sie die zutreffende Antwort für jeden Punkt aus:

	Variante A	Variante B	Keine der beiden Varianten
Eigentümerstruktur	Lohnunternehmer	Eigeninvestition	
Stickstoffeinsparung	-20%	-40%	
Unsicherheit	Auswirkungen während 5 Jahren<br>+ + + - +	Auswirkungen während 5 Jahren<br>+ 0 + 0 0	
Unterstützung	innerhalb 1 Stunde	am nächsten Tag	
Erhöhung Deckungsbeitrag	400 CHF/ha	200 CHF/ha und Jahr	0 CHF/ha und Jahr
	○	○	○

Eigentümerstruktur	Stickstoffreduktion	Unsicherheit	Unterstützung	Erhöhung Deckungsbeitrag
Eigeninvestition	Keine Reduktion	+ + 0 + 0	Keine Unterstützung	100 CHF/ha und Jahr
Lohnunternehmer	-20%	+ 0 + 0 0	innerhalb 1 Stunde	200 CHF/ha und Jahr
Gemeinsame Investition	-40%	+ + + - +	am selben Tag	300 CHF/ha und Jahr
		+ + + + +	am nächsten Tag	400 CHF/ha und Jahr
Der Landwirt kann entweder selbst in die Technologie investieren (Eigeninvestition), zusammen mit anderen Landwirten (gemeinsames Investition) oder die Leistung von einem Lohnunternehmer beziehen.	Reduktion der ausgebrachten Stickstoffmenge, ohne Ertragsverlust.	In wie vielen aus fünf Jahren bringt die Technologie einen Nutzen (+), keine Veränderung(0) oder einen Verlust (-)	Wie lange dauert es bis der Landwirt bei technischen Schwierigkeiten Unterstützung erhält.	Durch die Anwendung der Technologie entstehende höhere Deckungsbeiträge.

Können Sie erklären, warum Sie bei jeder Frage "Keine der beiden Varianten" gewählt haben? *

Bitte wählen Sie nur eine der folgenden Antworten aus:

- ◯ Ich denke, dass die aktuelle Situation gut genug ist.
- ◯ Ich halte teilflächenspezifische Stickstoffdüngnung nicht für wichtig.
- ◯ Sonstiges [ ]

II. Wahrnehmungen und Präferenzen

Im Folgenden möchten wir Ihnen einige Fragen zu Ihrer **persönlichen Wahrnehmung** und Ihren **Präferenzen** in Bezug auf die **Anwendung von teilflächenspezifischer Stickstoffdüngung** stellen. Es geht um **ihre ganz persönlichen Wahrnehmungen und Präferenzen** – es gibt also keine richtigen oder falschen bzw. besseren oder schlechteren Antworten. **Alle Antworten sind (wie bereits oben erwähnt) selbstverständlich strikt anonym und werden nur aggregiert ausgewertet.**

Haben Sie bereits Erfahrungen mit der Anwendung von teilflächenspezifischer Stickstoffdüngung? *

Bitte wählen Sie alle zutreffenden Antworten aus:

- ☐ Ja, ich verwende auf meinem Betrieb bereits teilflächenspezifische Stickstoffdüngnung.
- ☐ Ja, ich habe die Anwendung von teilflächenspezifischer Stickstoffdüngung bereits auf meinem Betrieb ausprobiert.
- ☐ Nein, aber ich habe Nachbarn/Freunde mit Erfahrungen.
- ☐ Nein, aber mein Berater kennt sich aus.
- ☐ Nein, und ich kenne auch niemanden persönlich mit Erfahrungen.
- ☐ Sonstiges: [ ]

Welche Präzisionstechnologien? *

Beantworten Sie diese Frage nur, wenn folgende Bedingungen erfüllt sind:
Antwort war 'Ja, ich habe die Anwendung von teilflächenspezifischer Stickstoffdüngung bereits auf meinem Betrieb ausprobiert. ' *oder* 'Ja, ich verwende auf meinem Betrieb bereits teilflächenspezifische Stickstoffdüngnung.' bei Frage ' [Q02]' (Haben Sie bereits Erfahrungen mit der Anwendung von teilflächenspezifischer Stickstoffdüngung?)

Bitte geben Sie Ihre Antwort hier ein:

[ ]

Bitte zählen Sie alle Technologien aus dem Bereich Präzisionslandwirtschaft auf.

II. Wahrnehmungen und Präferenzen

Bei der Anwendung von neuen Technologien oder technischen Problemen bevorzuge ich Unterstützung und Informationen… *

Bitte wählen Sie alle zutreffenden Antworten aus:

- ☐ von kantonalen Beratungsstellen
- ☐ von anderen Fachstellen (z.B. Agroscope)
- ☐ von Fachmedien (Zeitungen/Zeitschriften/Bücher)
- ☐ von Beratern der Technologiehersteller
- ☐ aus dem Internet (z.B. Blogs, Onlineforen etc.)
- ☐ von befreundeten Landwirten
- ☐ von benachbarten Landwirten
- ☐ Sonstiges: [ ]

Mit welchen Investitionskosten rechnen Sie bei der Anwendung von teilflächenspezifischer Stickstoffdüngung (CHF/ha und Jahr)? *

Bitte wählen Sie die zutreffende Antwort für jeden Punkt aus:

	0-50	51-100	101-150	151-200	201-250	251-300	301-350	351-400	401-450	451-500	mehr als 500
**Investitionskosten in CHF pro Hektare und Jahr**	◯	◯	◯	◯	◯	◯	◯	◯	◯	◯	◯

Die Erhöhung der Deckungsbeiträge durch die Anwendung der Technologie sollte basieren auf... *

Bitte wählen Sie alle zutreffenden Antworten aus:

- [ ] höheren Erträgen
- [ ] höhreren Produktpreisen
- [ ] Subventionen
- [ ] einer Labelprämie
- [ ] Sonstiges: ____________

Die folgenden Aussagen treffen auf mich und meinen Betrieb zu:
*

Bitte wählen Sie die zutreffende Antwort für jeden Punkt aus:

	Trifft nicht zu 1	2	3	4	Trifft vollkommen zu 5
**Die Anwendung einer teilflächenspezifischen Stickstoffdüngung wird positive Effekte für die Umwelt haben.**	○	○	○	○	○
**Mein Stickstoffeinsatz hängt vom Weizenpreis ab.**	○	○	○	○	○
**Ich habe den Anspruch Stickstoff so effizient wie möglich einzusetzen.**	○	○	○	○	○
**Die Anwendung einer teilflächenspezifischen Stickstoffdüngung wird positive Effekte auf die Gesundheit von Landwirte/innen und Konsument/innen haben.**	○	○	○	○	○
**Ich bin offen für landwirtschaftliche Innovationen und setze sie oft als Erste/r in der Region um.**	○	○	○	○	○
**Eine hohe Artenvielfalt auf meinen landwirtschaftlichen Flächen ist mir wichtig.**	○	○	○	○	○
**Ein möglichst hohes Einkommen aus der Produktion (inkl. Flächenbeiträge) zu erzielen, ist mir wichtig**	○	○	○	○	○
**Hohe Erträge (dt/ha) zu erzielen, ist mir wichtig.**	○	○	○	○	○
**Mein Stickstoffeinsatz hängt von den N-Dünger-Preisen ab.**	○	○	○	○	○
**Es ist wichtig, negative Auswirkungen der landwirtschaftlichen Produktion auf die Umwelt zu reduzieren.**	○	○	○	○	○
**Ich beschäftige mich gerne mit neuen Technologien und deren Anwendung.**	○	○	○	○	○
**Bei wichtigen landwirtschaftlichen Entscheidungen hole ich mir oft Rat bei meinen Nachbarn/Kollegen.**	○	○	○	○	○
**Wenn ich in der landwirtschaftlichen Produktion auf Schwierigkeiten stosse, kann ich normalerweise eine Lösung finden.**	○	○	○	○	○
**Entscheidungen von benachbarten Landwirten beeinflussen meine Anwendungsentscheidungen bei technischen Innovationen.**	○	○	○	○	○
**Die meisten technischen Probleme kann ich lösen, wenn ich mich anstrenge.**	○	○	○	○	○
**Der Erfolg in der landwirtschaftlichen Produktion hängt hauptsächlich von den Fähigkeiten des/der Landwirt/in ab.**	○	○	○	○	○
**Was meine Nachbarn und Freunde von mir denken, ist mir wichtig.**	○	○	○	○	○
**Der Erfolg in der landwirtschaftlichen Produktion kann nur in geringem Ausmass von Landwirten/innen beeinflusst werden.**	○	○	○	○	○

III. Risikoeinstellungen

Im Folgenden möchten wir Ihnen einige Fragen zu Ihren **Risikoeinstellungen** stellen. Es geht um **ihre ganz persönlichen Wahrnehmungen und Präferenzen** – es gibt also keine richtigen oder falschen bzw. besseren oder schlechteren Antworten. **Alle Antworten sind (wie bereits oben erwähnt) selbstverständlich strikt anonym und werden nur aggregiert ausgewertet.**

Wie beurteilen Sie Ihre persönliche Neigung, **Risiken** einzugehen? *

Bitte wählen Sie die zutreffende Antwort für jeden Punkt aus:

	Keine Risikobereitschaft 0	4	3	1	Sehr hohe Risikobereitschaft 10	2	9	8	7	6	5
**Generelle Risikobereitschaft**	○	○	○	○	○	○	○	○	○	○	○

Bitte geben Sie Ihre Präferenzen auf der Skala von 0 („nicht bereit Risiken einzugehen") bis 10 („sehr hohe Bereitschaft Risiken einzugehen") an. Mit den Zwischenwerten können Sie eine Tendenz in die eine oder andere Richtung angeben.

Wie beurteilen Sie Ihre persönliche Neigung, **Risiken** einzugehen in den **folgenden Bereichen**? *

Bitte wählen Sie die zutreffende Antwort für jeden Punkt aus:

	Keine Risikobereitschaft 0	4	3	1	Sehr hohe Risikobereitschaft 10	2	9	8	7	6	5
**Entscheidungen auf meinem Betrieb (allgemein)**	○	○	○	○	○	○	○	○	○	○	○
**Anwendung neuer Technologien**	○	○	○	○	○	○	○	○	○	○	○
**Landwirtschaftliche Produktion**	○	○	○	○	○	○	○	○	○	○	○

Bitte geben Sie Ihre Präferenzen auf der Skala von 0 („nicht bereit Risiken einzugehen") bis 10 („sehr hohe Bereitschaft Risiken einzugehen") an. Mit den Zwischenwerten können Sie eine Tendenz in die eine oder andere Richtung angeben.

Wie **wirtschaftlich riskant** schätzen Sie folgende Aspekte in Bezug auf die Investition in neue Maschinen für die teilflächenspezifische Stickstoffdüngung ein? *

Bitte wählen Sie die zutreffende Antwort für jeden Punkt aus:

	Kein Risiko 0	9	sehr hohes Risiko 10	1	2	3	4	5	6	7	8
**Investition kann nicht amortisiert werden**	○	○	○	○	○	○	○	○	○	○	○
**Maschinen verlieren durch technische Entwicklungen rasch an Wert**	○	○	○	○	○	○	○	○	○	○	○
**Erhöhte Fluktuation des Einkommens**	○	○	○	○	○	○	○	○	○	○	○

Wie stark tragen die folgenden Aussagen zu Ihrer Risikoeinschätzung einer Investition in Maschinen zur teilflächenspezifischen Stickstoffausbringung bei? *

Bitte wählen Sie die zutreffende Antwort für jeden Punkt aus:

	Nicht wichtig 1	2	3	4	Sehr wichtig 5
**Ich habe die Befürchtung, dass sich Direktzahlungsprogramme bald wieder ändern.**	○	○	○	○	○
**Ich habe die Befürchtung, dass die Preise für Ackerkulturen in Zukunft sinken.**	○	○	○	○	○
**Ich habe die Befürchtung, dass Lohnunternehmer den Service günstiger anbieten und meine Investition sich nicht lohnt.**	○	○	○	○	○
**Ich habe die Befürchtung, dass die Maschine zu wenig genutzt wird (Anschaffung nicht rentabel).**	○	○	○	○	○
**Ich habe die Befürchtung, dass die Technologie nicht zu höheren Deckungsbeiträgen führt.**	○	○	○	○	○

IV. Soziales Netzwerk

Mithilfe der folgenden Fragen möchten wir verstehen, welche Rolle soziale Beziehungen und Netzwerke bei der Anwendungsentscheidung von neuen Technologien, wie beispielsweise teilflächenspezifischer Stickstoffdüngung, spielen.

Gibt es Landwirte in ihrem Umfeld, welche teilflächenspezifische Stickstoffdüngung bereits anwenden? *

Bitte wählen Sie alle zutreffenden Antworten aus:

☐ Weiss nicht
☐ Ja
☐ Nein

Wie viele Landwirte in ihrem Umfeld wenden die Technologie bereits an? *

Beantworten Sie diese Frage nur, wenn folgende Bedingungen erfüllt sind:
Antwort war 'Ja' bei Frage ' [Q11]' (Gibt es Landwirte in ihrem Umfeld, welche teilflächenspezifische Stickstoffdüngung bereits anwenden?)

❶ In dieses Feld dürfen nur Zahlen eingegeben werden.
Bitte geben Sie Ihre Antwort hier ein:

V. Persönliche Eigenschaften BetriebsleiterIn und Charakteristika Betrieb

Um Unterschiede zwischen den Betrieben in unseren Ergebnissen zu berücksichtigen, wollen wir Sie im folgenden Teil der Umfrage ebenfalls über Ihre persönliche Eigenschaften - und Charakteristiken Ihres Betriebes befragen. Ihre Antworten werden **strikt anonymisiert und nur aggregiert** ausgewertet.

Bitte geben Sie ihr **Geburtsjahr** an (*z.B. 1963*): *

❶ Ihre Antwort muss zwischen 1919 und 2004 liegen.
Bitte geben Sie Ihre Antwort hier ein:

Welches ist die **höchste Ausbildung**, die Sie abgeschlossen haben? *

❶ Bitte wählen Sie eine der folgenden Antworten:
Bitte wählen Sie nur eine der folgenden Antworten aus:

○ Landwirtschaftliche Lehre (EFZ)
○ Landwirtschaftliche Meisterprüfung
○ Agrotechniker/in oder Agrokaufmann/frau (HF)
○ Fachhochschule
○ Universität oder ETH
○ Sonstiges

Ist Ihre **Betriebsnachfolge** bereits geregelt? *

❶ Bitte wählen Sie eine der folgenden Antworten:
Bitte wählen Sie nur eine der folgenden Antworten aus:

○ Ja
○ Nein
○ Unklar
○ Noch nicht relevant
○ Sonstiges

Möchten Sie eine Zusammenfassung der Umfrageergebnisse erhalten? *

Bitte wählen Sie nur eine der folgenden Antworten aus:

○ Ja
○ Nein

In welchem Kanton befindet sich Ihr Betrieb? *

❶ Bitte wählen Sie die zutreffenden Antworten aus:
Bitte wählen Sie alle zutreffenden Antworten aus:

☐ Bern
☐ Solothurn

Haben Sie noch einen abschliessenden Kommentar?

Bitte geben Sie Ihre Antwort hier ein:

Herzlichen Dank für Ihre Teilnahme!

Ihre Angaben und persönlichen Daten werden selbstverständlich nur anonymisiert verwendet und streng vertraulich behandelt.

Bei Fragen und Anregungen kontaktieren Sie bitte:

Karin Späti
Gruppe für Agrarökonomie und -politik (AECP)
ETH Zürich

kspaeti@ethz.ch

Sie können den Browser nun schliessen.

Mit besten Grüssen,

Karin Späti

Übermittlung Ihres ausgefüllten Fragebogens:
Vielen Dank für die Beantwortung des Fragebogens.
